# Supporting the development of Inuit children in urban environments: What are the needs of Inuit families living in southern Québec?

**DOI:** 10.17269/s41997-025-01041-5

**Published:** 2025-06-09

**Authors:** Lauriane Ouellet, Marie Grandisson, Christopher Fletcher

**Affiliations:** 1 Département de médecine sociale et préventive, Université Laval, Québec, QC Canada; 2 Centre interdisciplinaire de recherche en réadaptation et intégration sociale, Université Laval, Québec, QC Canada; 3 Axe santé des populations et pratiques optimales en santé, Centre de recherche du CHU de Québec, Université Laval, Québec, QC Canada; 4 École des sciences de la réadaptation, Université Laval, Québec, QC Canada

**Keywords:** Inuit, Urban environments, Healthy child development, Family needs, Community and institutional support, Inuit, Milieux urbains, Développement sain de l’enfant, Besoins des familles, Soutien communautaire et institutionnel

## Abstract

**Objectives:**

Canadian Inuit children present more developmental vulnerabilities than other non-Indigenous children. Supporting the development of these children is therefore essential, especially in urban environments where the Inuit population is growing. This study aimed to identify the main resources used by Inuit families living in urban environments of southern Québec, (Canada) and to better understand their needs related to supporting the healthy development of their children.

**Methods:**

A descriptive qualitative study was conducted using semi-structured interviews with 13 self-identified Inuit parents of at least one child aged 0 to 18 living in southern Québec.

**Results:**

The study revealed that urban Inuit families primarily require support to meet their basic needs, ensure a safe family environment, promote their children’s health, education, and socialization, foster the transmission of Inuit cultural and linguistic heritage, and, finally, enhance access to a culturally safe social environment. The study also revealed that despite living closer to a variety of resources intended to support their children’s healthy development, families encounter significant barriers in accessing these resources. Moreover, families have specific needs that are often not adequately addressed by the resources currently available.

**Conclusion:**

The study highlighted that the needs of Inuit families extend well beyond support for skills acquisition in the various developmental domains. Supporting the healthy development of Inuit children therefore requires a coherent and intensive response to families’ most urgent needs. In the light of such findings, there is a clear need to improve access to existing resources, as well as to develop Inuit-led services that are adapted to realities and specific needs of the families.

## Résumé

**Objectifs:**

Les enfants inuit canadiens présentent davantage de vulnérabilités développementales que les autres enfants non autochtones. Il apparaît donc essentiel de mieux soutenir le développement de ces enfants, notamment dans les milieux urbains où la population inuit est en croissance. Le but de cette étude était d’identifier les principales ressources utilisées par les familles inuit vivant dans les milieux urbains du sud du Québec (Canada) ainsi que de mieux comprendre leurs besoins en matière de soutien au développement sain de leurs enfants.

**Méthode:**

Une étude qualitative descriptive a été réalisée au moyen d’entrevues semi-structurées conduites auprès de 13 parents s’identifiant comme Inuit, ayant au moins un enfant âgé entre 0 et 18 ans et vivant dans le sud du Québec.

**Résultats:**

Cette étude a fait ressortir que les principaux besoins des familles inuit vivant en milieux urbains sont le soutien à la réponse aux besoins de base, à la mise en place d’un environnement familial sécuritaire, à la santé, à l’éducation et à la socialisation des enfants, à la transmission du patrimoine culturel et linguistique inuit et, finalement, à l’accès à un environnement social culturellement sécuritaire. Cette étude a également révélé que malgré la proximité d’une multitude de ressources visant à soutenir le développement sain de leurs enfants, les familles font face à de nombreux obstacles qui limitent leur accès à ces ressources. Par ailleurs, les familles présentent des besoins spécifiques auxquels il est plus difficile de répondre avec les ressources actuellement disponibles.

**Conclusion:**

Cette étude a permis de mettre en lumière que les besoins des familles inuit vont bien au-delà du soutien à l’acquisition d’habiletés dans les différentes sphères développementales. L’amélioration du soutien au développement sain des enfants inuit passe donc par une réponse cohérente et intensive aux besoins les plus criants des familles. À la lumière de ces résultats, il apparaît nécessaire d’améliorer l’accès aux ressources déjà existantes de même que de développer, en collaboration avec la communauté inuit urbaine, de nouveaux services adaptés aux réalités et aux besoins spécifiques des familles.

## Introduction

Children’s developmental health should be a societal priority (Irwin et al., 2007; Raphael et al., 2020). In fact, early childhood represents a crucial period in human development, during which experiences significantly influence long-term health and life trajectories (Nunavik Regional Board of Health and Social Services (NRBHSS), 2015). Supportive social environments, adequate living conditions, and access to high-quality health and education resources during early childhood have profound and lasting impacts throughout the lifespan (Irwin et al., 2007; Reading & Wien, 2009; NRBHSS, 2015).

Curently, Canadian Indigenous children, including Inuit children, face significant and wide-ranging health inequalities compared to their non-Indigenous peers (Halseth & Greenwood, 2019; NRBHSS, 2015). They are also at greater risk of developmental vulnerabilities and delays (Cappiello & Gahagan, 2009; Public Health Agency of Canada, 2018).

It is therefore essential to support programs that foster the care and healthy development of Indigenous children, as doing so can provide a better start in life and thus contribute to the long-term health and well-being of Indigenous communities (Halseth & Greenwood, 2019). However, in recent years, the chronic and discriminatory underfunding of services for First Nations children, including health and developmental support services, has been acknowledged in Canada (Blackstock, 2016). Gaps in both access to services and the quality of health care provided to Indigenous people have also been observed across the province of Québec and the rest of Canada (Commission d’enquête sur les relations entre les Autochtones et certains services publics au Québec (CERP), 2019; Truth & Reconciliation Commission of Canada, 2015). These challenges are also present in urban environments, where Indigenous peoples often come from distant and culturally distinct traditional territories, while available health services are typically designed for the non-Indigenous population, with limited adaptation to Indigenous sociocultural contexts and specific health needs (Collier, 2020; CERP, 2019). As a result, despite living closer to an array of health services, some Indigenous people living in urban environments face significant barriers to accessing appropriate care (Graham et al., 2023).

This situation is particularly concerning given the increasing number of Indigenous people living in urban environments across Canada (Statistics Canada, 2022). Inuit are no exception to this urbanization movement. In Québec, it is estimated that nearly 20% of Nunavik Inuit now live outside their traditional territory (Qanuikkat Siqinirmiut?, n.d.) with most living in urban environments (Statistics Canada, 2022). Montréal hosts the third largest Inuit population among Canadian cities, following Ottawa-Gatineau and Edmonton (Statistics Canada, 2022). While Statistics Canada (2022) report 1130 Inuit living in Montréal, a community-led census suggests that roughly twice that number are living in that city (Fletcher et al., 2022a). A similar undercounting has been reported in Ottawa-Gatineau (Smylie & Firestone, 2017). Despite the growing number of Inuit children and families living in urban environments, data and research on their realities remain scarce, and few services have been specifically designed to meet their needs (Qanuikkat Siqinirmiut?, n.d.; Smylie & Firestone, 2017).

In this context of a community that remains under-recognized and underserved, the aim of this study was to identify the main resources, or sources of support, that Inuit families living in urban environments of southern Québec draw upon to foster their children’s healthy development. The study also sought to gain a deeper understanding of the specific needs of these families in this regard.

## Methods

### Type of study and context

A qualitative descriptive approach (Sandelowski, 2000) was used to gather narrative data. This study was part of the Qanuikkat Siqinirmiut? (How are the people in the South?) project, community-based participatory research led by Christopher Fletcher in collaboration with the urban Inuit community and health organizations. The project aimed at building a knowledge base about Inuit living in southern Québec, in order to support the development of services both with and for this community. To this end, key members of the urban Inuit community were consulted at different stages to ensure that the study objectives and data collection method aligned with their priorities, values, and worldview. At the outset, the project concept was shared with and approved by these key members. In March 2022, a meeting was held with an Inuk woman who is deeply involved with the Inuit community of Montréal to better understand the specific realities and contexts of Inuit families living in southern Québec, and to learn about existing resources supporting Inuit child development. The study was approved by the *Comité d’éthique de la recherche avec des êtres humains de l’Université Laval* (Université Laval Human Research Ethics Committee).

### Theoretical frameworks

**Fig. 1:**
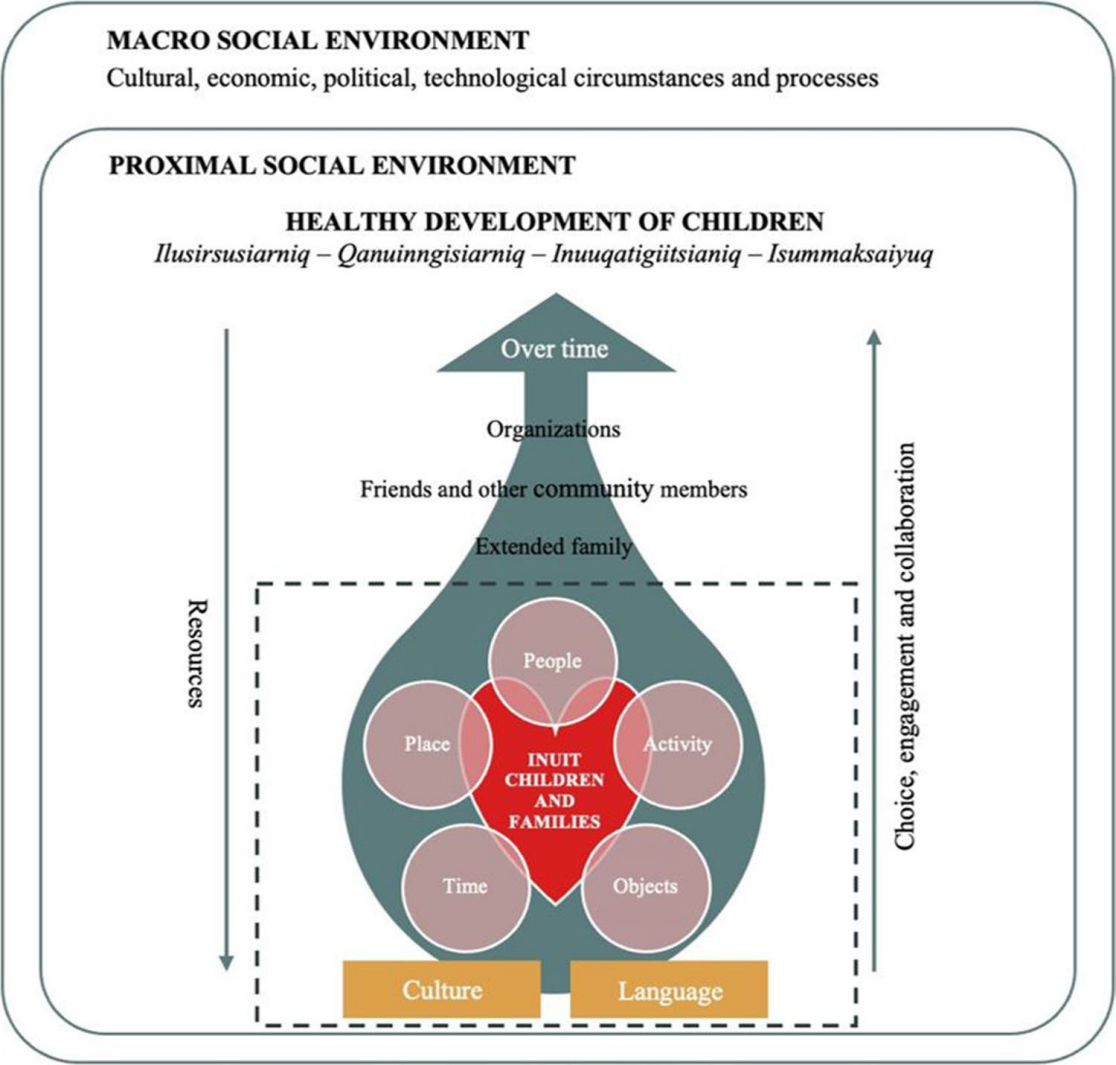
Integrative model

Three theoretical frameworks guided this study: the *Ilusirsusiarniq, Qanuinngisiarniq, Inuuqatigiitsianiq* (IQI) model (Fletcher et al., 2021); the child developmental approach of *Inunnguiniq* (Tagalik, 2010); and the Integrated model of social environment and social context for pediatric rehabilitation (Batorowicz et al., 2016). First, the IQI model consists of three interrelated concepts—*Ilusirsusiarniq, Qanuinngisiarniq, and Inuuqatigiitsianiq*—that together define health and well-being from an Inuit cultural and phenomenological perspective. The *Inunnguiniq* approach, for its part, describes the traditional process of socialization and education of Inuit children. These two theoretical frameworks were selected to ground the study in Inuit perspective of health, well-being, and child development. Finally, the Integrated model of social environment and social context for pediatric rehabilitation was used to explore how children interact with their social environment over time, and how this interaction shapes their development. This model supported an examination of the influence of the social environment on the experiences of Inuit children and families living in southern Québec. An integrative model was developed to bring together key elements from all three frameworks and to reflect the unique reality of Inuit child development (see Fig. 1).

### Recruitment and participants

This study sought to understand the perspectives of Inuit parents living in southern Québec. To recruit participants, a non-probability network sampling strategy was used (Patton, 2015). More specifically, the study was promoted on the Qanuikkat Siqinirmiut? project Facebook page, which was subsequently shared by individuals within the community network to broaden its reach. Additionally, a physical poster was displayed at a key Inuit community organization based in Montréal that serves as a gathering place for Inuit living in urban environments. To complement these efforts, a staff member from this organization personally reached out to individuals who met the inclusion criteria, providing information about the study and inviting them to participate. Interested individuals were then contacted by phone, email, or social networks by the first author or by another member of the Qanuikkat Siqinirmiut? project. To be eligible, participants had to (1) self-identify as Inuit; (2) be the parent of at least one child aged 0 to 18; (3) have lived in southern Québec for at least 6 months; and (4) be 16 years of age or older.

Free, informed, and ongoing consent of each participant was ensured throughout the study. Consent was obtained either in writing or verbally, depending on the participant’s preferences, in accordance with the guidelines for research involving Indigenous peoples in Canada (Canadian Institutes of Health Research et al., 2022). All participants received a modest financial compensation for their time.

### Data collection

Semi-structured individual interviews were conducted with the participants. To guide these discussions, an interview guide was developed by the research team, drawing on the integrative model presented above. A pretest interview was carried out with a participant who met the study’s inclusion criteria and had a good understanding of both the research area and the Qanuikkat Siqinirmiut? project. This pretest provided valuable feedback on the clarity, phrasing, and cultural relevance of the questions, allowing for adjustments to improve the interview process. As the revisions were minor, the transcript from this pretest interview was included in the data analysis. The interviews were conducted between March and June 2022 by the first author, a non-Inuit health professional who has been working with Nunavik Inuit communities for 5 years. They were held in English (*n* = 7) or French (*n* = 6) and were conducted virtually (*n* = 10) or in person (*n* = 3), depending on the participants’ preferences, geographical constraints, and public health measures in place during the COVID-19 pandemic. Each interviews lasted between 70 and 105 minutes, and was recorded and fully transcribed for analysis.

### Data analysis

Interview transcripts were analyzed using thematic-based content analysis (Paillé & Mucchielli, 2021). To this end, the first author segmented and grouped the data into categories using Nvivo software. Both inductive and deductive approaches (Hsieh & Shannon, 2005) guided the analysis: categories from the integrating model served as initial coding anchors, while new themes were created based on elements emerging from the analysis.

To ensure the credibility of results (Fortin & Gagnon, 2015; Kivits et al., 2016; Lincoln & Guba, 1986), external validation was carried out at various stages by individuals with relevant methodological or subject-matter expertise. First, a member of the Qanuikkat Siqinirmiut? project reviewed the coding of the first five interviews (38.5% of the corpus). Then, the co-authors, researchers with experience in child and family services and Inuit communities, carefully reviewed the study’s results, interpretations, and conclusions. Finally, preliminary findings were presented to key members of the urban Inuit community in September 2022, and the final results, interpretations, and conclusions were shared with them in April 2023. These meetings aimed to validate the results, correct any cultural inaccuracies, and ensure that the community’s knowledge was respected (Canadian Institutes of Health Research et al., 2022). 

## Results

### Participant profile

A total of 13 participants took part in the study. Their profiles varied in terms of age, number of children, family background, identity of their parents, place of origin of their biological Inuit parent(s), number of years spent in an urban environment, current place of residence, spoken languages, and occupation (see Table 1). The vast majority self-identified as female (*n* = 12) and lived in or near Montréal (*n* = 11). Additionally, just under a third (*n* = 4) were students, suggesting that some might only be in the city for the duration of their studies rather than settling permanently.

**Table 1:** Participant profile

		Number of participants (*n* = 13)
Age	16–24	1
	25–34	5
	35–44	5
	45–54	2
Gender	Woman	12
	Man	1
Number of children	1	4
	2	4
	3	3
	5	1
	7	1
Family background	In a relationship with the child(ren)’s other parent	3
	Sole custody of child(ren)	7
	Shared custody of child(ren)	3
Identity of their parents	Inuit parents	5
	One Inuk parent and one non-Inuk parent	7
	Non-Inuit adoptive parents	1
Place of origin of their biological Inuit parent(s)	Nunavik (Québec)	8
	Nunavut	3
	Newfoundland and Labrador	1
	Northwest Territories	1
Number of years spent in an urban environment	Less than 5	2
	5–9	2
	10–19	2
	20 years and over	7
Current place of residence	Montérégie	3
	Montréal	8
	Québec City	2
Spoken languages	English, French, and Inuktitut	2
	English and French	6
	English and Inuktitut	3
	English	2
Occupation	Student	4
	Employed	8
	Unemployed	1

### Resources currently used by families

This study identified the two main categories of resources used by Inuit families living in southern Québec to support the healthy development of their children.

1. Family, friends, and other community members

Many participants reported receiving support from family members, whether these family members live in the South or the North. Parents and siblings were most frequently mentioned as primary sources of support. In cases where immediate family members were not present, some participants said that they could rely on extended family such as cousins, aunts, and uncles. Beyond family, many participants also mentioned that they could rely on the support of friends and other members of their community, including co-workers, neighbours, babysitters, and Indigenous Elders. Finally, a few participants who were separated or divorced from the other parent noted that they could still count on that person, or that person’s family network, when needed.

2. Organizations

Participants also indicated that they were able to access support of organizations to help them in various ways. These include community organizations serving the general population, the broader Indigenous population, or the Inuit population. An overview of the mission of each community organization identified by participants is provided in Table 2. In addition, participants mentioned receiving support from both public and private health and social service organizations, as well as daycare centres, and schools serving the general population.

**Table 2:** Summary table of community organizations identified by participants

Organization name Location Website	Mission
Community organizations serving the Inuit population	
Ivirtivik Centre Verdun borough, Montréal http://ivirtivik.org	The Ivirtivik Centre’s mission is to support members of the Inuit community in preparing for the job market or returning to school by helping them strengthen their personal and professional skills.
Southern Québec Inuit Association (SQIA) Southwest borough, Montréal https://www.facebook.com/SQIA2017/	The SQIA is an association that represents Inuit living in southern Québec. Its mission is to promote their well-being, support cultural preservation, and foster community engagement.
Community organizations serving the Indigenous population	
Native Friendship Centre of Montréal (NFCM) Ville-Marie borough, Montréal https://nfcm.org	The NFCM’s mission is to promote, develop, and enhance the quality of life in Montréal’s urban Indigenous community.
Native Friendship Centre of Québec (NFCQ) Haute-Saint-Charles borough, Québec City http://www.caaq.net	The NFCQ’s mission is to provide a gathering place in Québec City that meets the cultural, material, and social needs of Indigenous people living in urban areas.
Native Montréal Southwest borough, Montréal https://nativemontreal.com/	The Native Montréal’s mission is to support the holistic health, cultural strength, and success of Indigenous families, individuals, and our community living in the greater Montréal area.
Native Women’s Shelter of Montréal (NWSM) Ville-Marie borough, Montréal https://www.nwsm.info/en/home	The NWSM’s mission is to provide a safe and supportive environment that strengthens cultural identity, self-esteem, and independence for Indigenous women and their children.
Mamuk Multi-Services Centre Charlesbourg borough, Québec City https://www.rcaaq.info/les-centres/quebec/	The Mamuk Multi-Services Centre’s mission is to provide services to the urban Indigenous population and to promote cultural vitality and stronger connections between communities.
Missinak Community House Charlesbourg borough, Québec City https://missinak.ca/	The Missinak Community House’s mission is to offer a welcoming space for women and their children, providing a safe and supportive environment that fosters protection, healing, and solidarity.
Community organizations serving the general population	
Head & Hands Côte-Des-Neiges – Notre-Dame-De-Grâce borough, Montréal https://headandhands.ca	Head & Hands strives to promote the physical and mental well-being of youth. Its approach is preventive, inclusive, non-judgemental, and holistic, with a fundamental commitment to providing a supportive environment for youth experiencing marginalization(s). It seeks to empower youth, and to facilitate social change based on the needs of youth within the community and society at large.
LGBT + Family Coalition Mercier – Hochelaga-Maisonneuve borough, Montréal https://familleslgbt.org/en/	The LGBT + Family Coalition is a community rights organization that advocates for the social and legal recognition of families that come under the umbrella of sexual and gender diversity.
Elizabeth House Côte-Des-Neiges – Notre-Dame-De-Grâce borough, Montréal https://maisonelizabethhouse.com	Elizabeth House is a rehabilitation centre offering a continuum of intervention and support services to families with children aged 0–5 years. Its mission is to support moms-to-be and moms by preparing them for the arrival of their baby and helping them understand their roles as parents.

### Family needs

The study also highlighted seven key needs of Inuit families living in southern Québec to support the healthy development of their children.

1. Support for basic needs

Most participants emphasized the importance of ensuring that their family’s basic needs such as food, housing, clothing, and other material and financial resources are met. However, they also described the challenges they face in this regards, often due to insufficient income or difficulties managing their finances. One participant highlighted that the independent living skills taught in the South, particularly around financial literacy, differ significantly from those taught in the North, making the transition to urban life especially challenging for newly arrived parents: “I thought, when I first moved here, I thought… I had this idea where it’s gonna actually be cheaper, but then, it wasn’t. That was fun! Because those are all things that people learn down here at a young age, but it’s not taught up north” (Participant [P] 1). Some participants therefore stressed the need for increased access to socio-professional integration services, as well as support in areas such as financial management, administrative procedures, and acquiring the skills necessary for autonomous living in an urban environment. As this lack of income and difficulties in managing finances often lead to food and material insecurity within households, some expressed a desire to see improvements to the existing food aid program such as grocery store gift cards and Christmas baskets. Others suggested the creation of clothing donation services. Participants also pointed out that discrimination in the rental market can make it difficult for some members of their community to find housing. As such, access to support in finding housing was a desire identified by some participants. Finally, a number of participants mentioned struggling to find someone to look after their children, which limited their personal and/or professional opportunities. This challenge was especially acute for single parents with limited support networks, and some expressed a wish for improved access to babysitting services.

2. Support for a safe family environment

Creating a safe home environment was also crucial to many participants, as illustrated by the following quote: “Child development? I think the first base is to have a safe home environment” (P1). However, some participants reported experiencing or having experienced difficulty establishing such an environment, often due to challenges in developing and maintaining healthy relationships with their children, or because of violence or problematic drug or alcohol use in the home. Several participants linked these difficulties to the dislocation of Inuit through colonialism and the resulting intergenerational trauma: “Stepping back and trying to break that generational curse is hard. Very, very hard. That’s something I had to learn. […] It’s very important especially with the lifestyle that we have unfortunately succumbed to, especially with the colonization, how it affected us a lot” (P1). A number of participants expressed a desire to have access to more psychological services to support healing and the creation of a safer family environment. In particular, some called for Inuit-specific psychological services. Others emphasized the need for greater parenting support, such as workshops to build and strengthen parenting skills, and a support group for Inuit parents living in urban environments.

3. Support for children’s health

Many participants emphasized the importance of caring for their children’s physical and mental health. However, some expressed a lack of knowledge about how to support their children’s development, while others spoke of their difficulties accessing the health and social services that could help. Long wait times and challenges in having their children’s eligibility recognized for certain public programs or insurance plans were common concerns. Many participants wished their children to have easier access to services such as medical services and psychological support. Again, some also voiced a desire for more Inuit-specific psychological services. In addition, certain participants reported not knowing what resources were available or how to navigate the health and social services system: “When I first moved here, […] I didn’t know a lot of organizations, I didn’t know who to talk to. So, finding out where to bring my daughter to the clinic. Where to… Who to talk to when I felt like I was worried, but not worried enough to bring her to the hospital when she was sick” (P1). Finally, some mentioned transportation as a barrier to accessing services, citing unfamiliarity with the urban transit system, long distances to travel, or the cost of public transportation as limiting factors.

4. Support for children’s education

Participants consistently emphasized the importance of education for their children: “Going to school, education, is really important for me. You know, I want them [participant’s children] to go far in life in what interests them” (P3, our translation). While most held positive aspirations, certain participants shared concerns about their children’s difficulties at school. Those who had recently moved to an urban environment noted the challenges of transitioning between education systems, highlighting the educational gap between the North and the South: “She [participant’s daughter] was behind because of the educational gap that we have” (P1). In addition, some participants complained about the lack of pedagogical support available in their children’s school and expressed a desire for more resources to support their academic success and development, such as tutoring and homework assistance. Others pointed to a broader lack of services tailored to Indigenous students in the southern schools. Some wished to see more initiatives like access to Indigenous resource teachers, which is available in certain other regions of the country.

5. Support for children’s socialization

For many participants, it was important to give children opportunities to socialize and build relationships with a variety of people, including relatives, members of the Inuit or broader Indigenous community, and non-Indigenous individuals. However, some described their difficulty in supporting their children’s socialization due to the social isolation their families experience. This challenge appeared particularly significant for newly arrived families and those with only one child: “To do activities, it would always end up being just me and her [participant’s daughter], which was kind of isolating in the beginning” (P1). Certain participants expressed a desire to have access to more resources to support their children’s socialization. In particular, some highlighted the importance of creating opportunities for their children to connect with other members of the urban Inuit community, including other children and Elders.

6. Support for the transmission of cultural and linguistic heritage

The transmission of Inuit cultural and linguistic heritage to children was of vital importance for many participants. One key aspect of this heritage is access to traditional foods. However, some reported difficulties in accessing “country food”^1^ and in engaging in traditional Inuit activities such as hunting, fishing, and berry-picking. These barriers hinder their ability to teach their children traditional skills: “And as for hunting, I cannot because… I don’t even know if we are even allowed. And even if we are allowed, I would never want to fish in your river here. It might be full of pollution or something. […] I’m not able to teach that here” (P11). A number of participants expressed a desire for their children to have greater access to Inuit-specific cultural activities, such as community feasts, sewing, beadwork, and traditional music, as well as Inuit games.^2^ Others highlighted the need for resources to support Inuktitut language learning, noting the difficulty of passing on the language due to limited opportunities to speak it in their community and, in some cases, the fact that many of them speak little or no Inuktitut. Some also indicated that they would like to participate in these activities themselves, to strengthen their own knowledge and better transmit it to their children.

7. Access to a culturally safe social environment

Many participants emphasized the importance for families of living within a culturally safe social environment. However, several reported facing significant barriers to accessing such an environment, often due to experiences of discrimination and communication barriers in their interactions with others: “Because you have a language barrier, you stay to yourself, you don’t want to go out there because you’re afraid of being judged and racism” (P6). Some participants also deplored the lack of recognition for Inuit culture and language. For instance, three mentioned that they had been advised by certain professionals to forgo transmitting Inuktitut to their children in favour of English or French. Others expressed concern about the limited understanding among some service providers of Inuit social, cultural, and historical realities. In light of these experiences, many participants expressed a desire for services specifically tailored to Inuit population, highlighting the linguistic and cultural distinctiveness of Inuit compared to other Indigenous groups. A number of participants also expressed a desire to have access to a dedicated space where they could connect with other members of the urban Inuit community and receive various forms of support: “It’s gonna help so much just to be able to have a place to go and ask about anything. […] You know, I need help about this. How do I learn how to do this? Just information. […] I think it would definitely help parents get together, because when we see Inuit, we enjoy. We have a place to go” (P1). In addition, many participants expressed a strong preference for receiving services from Inuit workers. In the absence of Inuit workers, some emphasized the importance of non-Inuit workers demonstrating knowledge of and genuine respect for Inuit culture: “If it couldn’t be Inuit […], I would say knowledgeable individuals who have good knowledge of our culture, and respect of our culture because you could have knowledge, but you don’t necessarily have respect […] So, I guess in a sense, like a really good ally. […] True allyship and very, very little judgment” (P5). Finally, some called for interpreter services to support parents who do not speak English or French, as well as tutoring services to help community members learn one of those languages and reduce communication barriers.

## Discussion

This study showed that supporting the healthy development of Inuit children living in the urban environments of southern Québec requires much more than fostering skill acquisition in various developmental domains. Families primarily require support to meet their basic needs, ensure a safe family environment, promote their children’s health, education, and socialization, foster the transmission of Inuit cultural and linguistic heritage, and, finally, enhance access to a culturally safe social environment. These findings are consistent with those of Reading and Wien (2009) who emphasize that fostering Indigenous children’s development requires action on the proximal-level social determinants of health such as the physical and social environment and food security, as well as on intermediate and distal-level determinants such as health and education systems, cultural continuity, social cohesion, colonialism, social exclusion, repression of self-determination, and racism. In the light of such findings, it is clear that without an appropriate response to these needs and action on the associated social determinants of health, services aimed at supporting the development of Inuit children of southern Québec risk falling short of what families truly require.

The study also highlighted that Inuit families living in southern Québec can rely on their extended family, friends, and other community members to support their children’s healthy development. This finding aligns with that of Fletcher et al. (2022a). who emphasize that mutual social support is a key element of community resilience among Inuit living in the South. In addition, the study showed that Inuit families living in southern Québec receive support from a range of organizations, including community organizations, health and social services clinics and organizations, daycare centres, and schools.

However, our results indicate that these families face a range of barriers when trying to access the services offered by some of these organizations. Many of these barriers have also been documented in the literature, including a lack of awareness of existing resources (Fletcher et al., 2022a; Patrick & Tomiak, 2008); wayfinding difficulties linked to navigating the health and social services system or understanding how it functions (CERP, 2019); transportation challenges (Graham et al., 2023; Patrick & Tomiak, 2008; Smylie & Firestone, 2017); communication and intercultural barriers (Graham et al., 2023; Patrick & Tomiak, 2008; Smylie & Firestone, 2017); and difficulties obtaining recognition of eligibility for certain public programs or insurance plans (CERP, 2019). It is therefore essential to improve access to information about available services, a need that has been voiced by many Inuit living in southern Québec according to Fletcher et al. (2022a). The development of navigation and accompaniment services also appears to be an appropriate step to help families access these organizations, exercise their rights, and foster communication with community stakeholders. Such measures have also been recommended by the CERP (2019), Fletcher et al. (2022a); and Smylie and Firestone (2017). The latter two authors point out that Inuit-specific navigation services should be developed, as no such services currently exist for the urban Inuit community.

Moreover, several participants reported experiencing racial discrimination in their social environment, which has also been documented in other studies (Fletcher et al., 2022a; Graham et al., 2023; Patrick & Tomiak, 2008; Smylie & Firestone, 2017; Vaudry, 2016). Other participants spoke of their difficulties in accessing culturally safe services or in finding service providers with both knowledge of and experience working with the Inuit community. Similar findings were highlighted by Fletcher et al. (2022a) and Smylie and Firestone (2017). These results underscore the importance of developing training programs, co-created with the Inuit community, that aim to foster cultural awareness, sensitivity, and competence among service providers likely to interact with members of this community (Fletcher et al., 2022a; Fraser et al., 2021; Graham et al., 2023; Ouellet et al., 2022; CERP, 2019; Smylie & Firestone, 2017). As noted by Fletcher et al. (2022a), such training should specifically address the realities of urban Inuit, as the issues experienced by this population subgroup differ from those of other Inuit and urban Indigenous populations.

The study also showed that Inuit families living in southern Québec have specific needs that may be more difficult to meet with the community and institutional resources currently available. Many participants expressed a desire for more services tailored to the Inuit population, highlighting the potential value of a multiservice centre for Inuit living in southern Québec. In exploring options, the Tungasuvvingat Inuit (2020) and Inuuqatigiit Centre for Inuit Children, Youth, and Families (n.d.), both providing essential services to Ottawa’s urban Inuit population, could serve as valuable models for developing similar initiatives in Québec. The study has stressed the need for such a centre to develop services specifically for Inuit children and their families. Moreover, as the provision of more culturally safe psychological support for children and their parents as well as the opportunity to take part in activities to promote Inuit culture and language were identified as particularly important by participants, it would be particularly relevant for the centre to develop this type of service. These findings are consistent with previous research showing that Inuit living in urban environments benefit from increased access to mental health resources and stronger connections to Inuit culture and community (Fletcher et al., 2022a; Graham et al., 2023). To facilitate families’ engagement, participants also expressed a need for complementary supports such as transportation options, online or home-based services, and babysitting services. Finally, the study also highlighted the desire of many parents for service providers to be Inuit, or at least to have direct knowledge of and genuine respect for Inuit and their culture. This aligns with the recommendation of Fletcher et al. (2022a) to prioritize the hiring of Inuit or non-Inuit employees who are properly trained to work with this community. Beyond Inuit involvement in service delivery, several authors have stressed the importance of involving Indigenous communities in the planning, development, implementation, and evaluation of programs and services that concern them (Ball, 2005; Halseth & Murdock, 2020; Smylie et al., 2011). To this end, collaborations with non-Inuit with an interest in Inuit health or, more broadly, Indigenous health, would be important in order to benefit from their expertise (Halseth & Murdock, 2020). In this regard, it is recognized that non-Inuit can contribute greatly to the empowerment and self-determination of the Inuit community, as long as their practices and approaches respect and promote Inuit ways of knowing and doing (Fraser et al., 2021). Please see Table 3 for a summary of results and proposed courses of action.

**Table 3:** Summary of results and proposed courses of action

Family needs	Main resources used by families	Services requested by families	Main issues identified	Proposed courses of action
1. Support for basic needs	• Extended family, friends, and other community members: -Babysitting -Material and financial assistance -Support for day-to-day management -Housing • Organizations: ▪ Community organizations: -Socio-professional integration support** -Food and material assistance** -Support with financial management and administrative procedures* -Support in housing search* -Housing* ▪ Health and social services clinics and organizations: -Support with financial management and administrative procedures -Support for day-to-day management -Support in housing search ▪ Daycares and schools -Childcare	• Socio-professional integration support • Support with financial management and administrative procedures • Support in developing skills for independent living in urban environments • Support in housing search • Food assistance • Free babysitting services • Clothing exchange or donation services	• Limited awareness of available resources • Barriers to accessing existing resources	1. Improve access to information about available organizations and services to encourage greater use of existing resources. 2. Develop Inuit-specific wayfinding and support services to assist families in navigating various organizations and accessing the services they need. 3. Develop training programs tailored to service providers working with the urban Inuit community, aimed at fostering awareness, sensitivity, and cultural competence. 4. Plan the creation of a multi-service centre for Inuit living in southern Québec, offering services specifically for children and their families, including culturally safe psychological support and activities that promote Inuit culture and Inuktitut. 5. Improve access to services dedicated to the urban Inuit community by providing transportation options, online or in-home services, and babysitting services. 6. Promote Inuit participation in the planning, implementation, delivery, and evaluation of Inuit programs and services intended for them, ensuring meaningful involvement at every stage.
2. Support for a safe family environment	• Extended family, friends, and other community members: -Emotional support (for parents) -Informal support group for urban Inuit parents • Organizations: ▪ Community organizations: -Psychological support (for parents)* -Support for the development or reinforcement of parenting skills -Structured support group for parents -Support in navigating the legal system, advocating for families, and taking steps to prevent or end child placement (wayfinding)* ▪ Health and social services clinics and organizations: -Psychological support (for parents) -Child protection services	• Psychological support tailored to Inuit parents • Support for the development and reinforcement of parenting skills • Structured support group for urban Inuit parents	• Lack of psychological support tailored to Inuit parents • Absence of a structured support group for urban Inuit parents	
3. Support for children’s health	• Extended family, friends, and other community members: -Tips on how to care for and stimulate children’s development -Emotional support • Organizations: ▪ Community organizations: -Psychological support* ▪ Health and social services clinics and organizations: -Psychological support -Medical support -Development support ▪ Daycares and schools -Psychological support -Emotional support -Development support	• Psychological support tailored to Inuit children • Medical support tailored to Inuit children	• Lack of medical support tailored to Inuit children • Lack of psychological support tailored to Inuit children	
4. Support for children’s education	• Organizations: ▪ Community organizations: -Tutoring and homework assistance services* ▪ Daycares and schools: -Educational support -Adapted classes	• Tutoring and homework assistance services • Indigenous resource teachers	• Limited awareness of available resources • Barriers to accessing existing resources	
5. Support for children’s socialization	• Extended family, friends, and other community members: -Activities with family and friends • Organizations: ▪ Community organizations: -Activities with other members of the urban Inuit community** ▪ Daycares and schools -Activities with other children	• Activities with other members of the urban Inuit community, including other children and Inuit Elders	• Limited awareness of available resources • Barriers to accessing existing resources	
6. Support for the transmission of Inuit cultural and linguistic heritage	• Extended family, friends, and other community members: -Discussions in Inuktitut -Traditional Inuit or, more broadly, Indigenous activities Country food • Organizations: ▪ Community organizations: -Traditional Inuit** or, more broadly, Indigenous* activities -Community feasts** -Inuktitut classes*	• Traditional Inuit activities • Resources for learning Inuktitut	• Limited awareness of available resources • Barriers to accessing existing resources	
7. Access to a culturally safe social environment	• Extended family, friends, and other community members: -Accompaniment to certain appointments to act as interpreter • Organizations: ▪ Community organizations: -Support in navigating the health and social services system, advocating for families, and facilitating communicating with various service providers (wayfinding)* ▪ Daycares and schools: -Presentation on Inuit culture in schools	• Services specifically tailored to the urban Inuit community • Services offered by Inuit workers or non-Inuit workers with knowledge of and respect for Inuit culture • Interpreter services • English and/or French tutoring services for Inuit parents	• Lack of Inuit-specific services • Lack of Inuit service providers within organizations • Limited understanding of Inuit realities among non-Inuit service providers	

*Services offered by organizations serving the Indigenous population

**Services offered by organizations serving the Inuit population

Finally, with regard to theoretical frameworks, the creation of an integrative model combining key elements of three theoretical frameworks (i.e. the IQI model, the child developmental approach of *Inunnguiniq,* and the Integrated model of social environment and social context for pediatric rehabilitation) proved to be highly useful in representing the unique reality of Inuit child development. Drawing on concepts that align more closely with Inuit worldview of child health and development was essential to moving beyond developmental perspectives rooted in Western paradigms. In this regard, some authors have stressed the importance of questioning conventional definitions of “health” and “research” when addressing Inuit health and well-being (Healey & Tagak Sr., 2014). Indeed, incorporating Inuit perspectives is essential to decolonizing and ensuring greater equity in the health services offered to this population (Fletcher et al., 2022b). In summary, this integrative model may serve as a valuable tool for future research on Inuit child development, whether in the South or North of the country. However, it is essential to adopt a sensitive, thoughtful, and flexible approach when applying such a model, as Inuit communities may hold diverse and evolving views.

### Study strengths and limitations

First, given the context of the COVID-19 pandemic, the challenge of engaging this specific population due to its geographic dispersal across a vast area, and other logistical constraints, the results of this study are based on interviews with 13 participants, a relatively small sample. Future studies could therefore benefit from integrating participant observation and conducting more, and more varied, interviews with service providers and other community members, such as Elders and youth groups.

Second, it was not possible to recruit qualified interpreters to conduct interviews in Inuktitut, as the few interpreters associated with the Qanuikkat Siqinirmiut? project were unavailable during the data collection period. Despite this, we are confident in the quality of the data, as nearly all participants were fully fluent in English or French. Only one participant reported feeling less comfortable expressing herself in one of these languages during the interviews.

Finally, it is important to acknowledge that the three authors of this article are not Inuit. To address this limitation, the research team ensured that each stage of the study, as well as the main findings, were validated by key members of the urban Inuit community, in order to align the research objectives, data collection method, and results with the community’s values, vision, and knowledge. In addition, the first author adopted a reflexive approach throughout the research process, notably through in-depth reflection on her own background and prior experiences (Ouellet et al., 2022). This strategy enabled her to better understand the influence of her own cultural and experiential biases on the research process, which is essential to ensuring both the quality and cultural relevance of research conducted in Indigenous contexts (Nilson, 2017). Moreover, two of the three authors have professional experience and prior knowledge of Inuit realities, which can contribute to the development of a culturally relevant research protocol, as well as a more contextualized interpretation of participants’ narratives (Bird et al., 2009).

## Conclusion

This study revealed that the needs of Inuit families living in urban environments of southern Québec, in terms of support for their children’s healthy development, span multiple aspects of their lives. Addressing these needs requires first and foremost a recognition and deeper understanding of the unique socio-historical conditions that contribute to the inequities experienced by Inuit families. The challenges participants identified in this study are rooted in these historical events, and addressing the social determinants of health at all levels will help mitigate these challenges. To achieve this, existing services must become more accessible, notably by improving access to information about relevant organizations and services in existence, by developing Inuit-specific navigation services, and by offering training for service providers working with members of the urban Inuit community. Ultimately, the development of health and social services led by and for Inuit, alongside support for community-driven initiatives such as a cultural community centre, are essential to create environments where children and families can thrive.

## Contributions to knowledge

What does this study add to existing knowledge?

- To the best of our knowledge, this is the first study to examine the specific situation of Inuit children and their families living in urban environments of southern Québec.
- The needs of Inuit families living in southern Québec in terms of supporting healthy child development extend far beyond the acquisition of skills in the various developmental domains.
- Access to greater culturally safe psychological support for both children and parents, along with opportunities to participate in activities that promote Inuit culture and language, emerged as particularly important.
- Despite living in closer proximity to a range of child development resources, Inuit families living in southern Québec face several barriers that limit their access to these resources, and some of their specific needs remain unmet by the resources currently available.

What are the key implications for public health interventions, practice, or policy?

- The study underscored that improving support for the healthy development of Inuit children living in southern Québec requires, above all, a stronger response to families’ needs, which extend far beyond support for the acquisition of skills in the various developmental domains, and coordinate action on the social determinants of health at all levels that shape those needs.
- It also pointed to the importance of improving access to existing services, which could be achieved by developing Inuit-specific navigation services, improving access to information about relevant organizations, and providing training for service providers working with members of the urban Inuit community.
- The study also highlighted the need to develop services specifically tailored to Inuit families living in southern Québec, in partnership with the members of this community.

## Data Availability

Upon reasonable request.

## Footnotes

1 Country food refers to traditional Inuit food obtained through hunting, fishing, and gathering. Beyond its nutritional value, country food holds deep cultural and identity significance for the Inuit (Robinson, 2018).

2 Inuit games are traditional sporting activities designed to develop and maintain the physical and mental skills essential for survival in the Arctic. They are also an opportunity for enjoyment and celebration. These games continue to hold significant meaning among Inuit today (*Canadian Geographic*, n.d.).

## Code availability

Upon reasonable request.
